# Maintenance Treatment with Trofosfamide in Patients with Primary Bone Ewing Sarcoma – Single Center Experience

**DOI:** 10.34763/devperiodmed.20192301.3944

**Published:** 2019-04-08

**Authors:** Anna Raciborska, Katarzyna Bilska, Carlos Rodriguez-Galindo

**Affiliations:** 1 Department of Oncology and Surgical Oncology for Children and Youth, Institute of Mother and Child, Warsaw, Poland; 2 Departments of Global Pediatric Medicine and Oncology, St. Jude Children's Research Hospital, Memphis, TN, US

**Keywords:** bone Ewing sarcoma, trofosfamide, maintenance treatment, mięsak Ewinga, trofosfamid, leczenie podtrzymujące

## Abstract

**Background:**

Patients with Ewing sarcoma have a dismal outcome. Maintenance treatment with trofosfamide has been proposed as an effective regimen for same paediatric malignancies.

**Aim:**

We sought to evaluate the schedule of trofosfamide for patients with high-risk primary bone Ewing sarcoma.

**Materials and methods:**

Fifteen patients with primary bone Ewing sarcoma received treatment with trofosfamide (750 mg/m^2^ p.o. days 1-10) every 28 days. All patients hod standard tumour imaging and laboratory evaluation. All toxicities were documented.

**Results:**

A tatal of 90 cycles (median 5 cycles/patient) were administered. A complete response was maintained in nine patients, while six patients had disease progression during treatment Median time to progression was 7.9 months (range 1.8 to 4.6). Eleven patients (73.3%) are alive including nine with no evidence of disease with a median follow-up of 3.9 years (range 7.4 to 7.6). All patients with active disease at the start of the trofosfamide treatment died. There were no significant toxicities.

**Conclusions:**

Treatment with trofosfamide is well-tolerated and could have a role to maintain response in patients with primary bone Ewing sarcoma. Further studies are needed to better define the use of this regimen in the upfrront management of those patients.

## Streszczenie

**Wprowadzenie:**

Przebieg mięsaka Ewinga u części pacjentów jest niekorzystny. Wg niektórych doniesień trofosfamid może być skuteczny w leczeniu podtrzymującym wybranych nowotworów u dzieci.

**Cel pracy:**

Celem pracy była ocena schematu leczenia trofosfamidem pacjentów z kostną postacią mięsaka Ewinga z grupy wysokiego ryzyka.

**Materiały i metody:**

U 15 pacjentów z kostną postacią mięsaka Ewinga zastosowano trofosfamid (750 mg/m^2^ p.o. dzień 7-70) w schemacie co 28 dni. Wszyscy pacjenci mieli wykonane standardowe badania obrazowe oraz laboratoryjne. Prowadzono dokumentację wszystkich występujących objawów niepożądanych.

**Wyniki:**

W sumie przeprowadzono 90 cykli leczenia (z medianą 5 cykli u każdego pacjenta). U dziewięciu terapia trofosfamidem pozwoliła utrzymać całkowitą odpowiedź na zastosowane leczenie. U sześciuchorych stwierdzono progresję. Mediana czasu do progresji wyniosłą 1,9 miesiąca (zakres 1,8 do 4,6). 11 (73.3%) pacjentów żyje, w tym 9 bez objawów choroby, z medianą obserwacji 3,9 lat (zakres 1,4 do 7,6). Wszyscy pacjenci z aktywną chorobą, u których włączono trofosfamid, zmarli. W trakcie leczenie nie obserwowano istotnych toksyczności.

**Wnioski:**

Trofosfamid jest dobrze tolerowany u pacjentów z kostną postacią mięsaka Ewinga. Wydaje się, że mógłby być wykorzystany w utrzymaniu odpowiedzi na leczenie u tego typu chorych. Jednak, aby zastosować ten schemat jako rutynowe postepowanie, konieczne jest przeprowadzenie dalszych badań.

## Introduction

Ewing sarcoma is a small round blue cell tumour with varying degrees of neuroectodermal differentiation and a wide spectrum of clinical presentation, accounting for approximately 40% of all bone malignancies in children and young adults and 3% of soft tissue sarcomas. With advances in multimodal therapy, survival rates for patients with primary localized bone disease approach 70% to 75% [1, 2, 3]. However, patients with metastatic, progressive or recurrent disease have a dismal outcome [4, 5, 6, 7, 8].

The main rationale for administration of maintenance treatment in oncology is to preserve the remission and prevent recurrence of the disease, or to provide disease stabilization as a part of palliative treatment. While the role of maintenance treatment has been well established for pediatric hematological malignancies, its use in solid tumors, particularly sarcomas, is more controversial [9].

Trofosfamide has been proposed as an effective drug for some paediatric malignancies, particularly soft tissue sarcomas [10, 11, 12, 13, 14]; its use as oral agent in maintenance therapy has been proposed for patients with metastatic rhabdomyosarcoma [9]. Trofosfamide is a cyclophosphamide analogue alkylating agent; compared to the other drugs from that group trofosfamide is more lipophilic and available only as an oral formulation. The moderate toxicity profile observed in a few studies allows the consideration of trofosfamide as a reasonable maintenance treatment option also for heavily pretreated patients with sarcoma [9, 15, 16, 17].

## Aim of study

The aim of study was to evaluate the schedule of trofosfamide for patients with high-risk primary bone Ewing sarcoma.

## Materials and methods

### Patients

Fifteen patients with histologically confirmed primary high-risk bone Ewing sarcoma (ES) (4 primary metastatic, 11 recurrent) were treated with maintenance oral trofosfamide during the period 2012-2016 at Mother and Child Institute (Warsaw, Poland). Informed consent was obtained from all patients or their guardians before treatment. Approval for this retrospective study was obtained in compliance with international regulations for protection of human research subjects.

### Treatment

Trofosfamide was administered at a dose of 150 mg/ m2 divided into two doses for ten consecutive days. The cycles were repeated every 28 days. Treatment was to be continued for one year (12 cycles) or until disease progression or unacceptable toxicity. Patients developing myelosuppression were treated with granulocyte colonystimulating factor (G-CSF) and transfusion of blood products as clinically indicated. Dose reduction was undertaken in case of prolonged leukopenia despite the use of G-CSF or thrombocytopenia <75.000 for more than 2 weeks.

### Assessment of response and toxicity

All patients had standard tumour imaging using CT, MRI, bone scan or PET, as indicated, prior to starting trofosfamide and every three courses. Physical examination and laboratory evaluation were performed prior to each cycle or weekly when necessary. All toxicities were documented from day 1 of the first cycle until end of therapy. The WHO criteria were used to evaluate response.

### Methods

Overall Survival (OS) was defined as the time interval from the date of diagnosis to the date of death or to last follow-up date. Time to relapse was defined as the time interval from date of initial biopsy to date of disease recurrence. Time to progression was defined as the time interval from date of initial biopsy to date of disease progression. Trofosfamide OS was defined as the time interval from first day of trofosfamide treatment to the date of death or to last follow-up date. Trofosfamide time to progression (TTP) was defined as the time interval from start date of trofosfamide to date of disease progression. Results distributions were estimated using the method of Kaplan-Meier. Statistical analysis was performed using STATA 10.0 for Windows.

## Results

Between 2012 and 2016, 15 patients (8 males, 7 females) with histologically confirmed primary bone Ewing sarcoma were treated with oral trofosfamide. Patient characteristics and prior treatment are summarized in table I. Median age was 12.4 (range 2.6 to 19.2 years). Eleven patients had metastatic disease at diagnosis (nine of them only to the lung, two of them to the lungs and bones), eleven had recurrent disease, one patient had progression. Fourteen patients had received neoadjuvant chemotherapy according to the Euro Ewing protocol using the VIDE regimen (vincristine, doxorubicin, ifosfamide, etoposide) [18] and one had been treated according to CAV/ETIF regimen (alternating cycles of vincristine/cyclophosphamide/actinomycin/adriamycin and ifosfamide/etoposide). Local control included surgery in six patients, radiation therapy in three patients, and combined surgery and radiation in six patients. Following local control, patients received vincristine, dactinomycin, and cyclophosphamide/ifosfamide (VAI/VAC), depending on response. Seven patients received consolidation with high-dose chemotherapy and autologous hematopoietic stem cell transplant (HSCT). Eleven patients had experienced relapse, one had disease progression. Median time to relapse or progression from initial therapy was 9.9 months (range 2.5 to 52.8 months). Due to relapse or progression nine patients received VIT (vincristine, irinotecan, temozolomide) and three received the PACE (cisplatin, teniposide, adriamycin, cyclophosphamide) to induce a new response.

**Table I:** Patient and treatment characteristics at time of original diagnosis (n=15).

Gender	N%
Male	8 (53,3%)
Female	7 (46,7%)
Median age in years	12.4
Primary tumor location	
Extremity	9 (60%)
Axis	6 (40%)
Metastasis at initial diagnosis	11
Site of metastases at diagnosis	
Lungs	9 (81,8%)
Lungs + Bones	2 (18,2%)
Local treatment at diagnosis	
Surgery only	6 (40%)
RTX only	3 (20%)
Both	6 (40%)
Neoadjuvant chemotherapy	
VIDE regimen	14 (93,3%)
CAV/ETIF regimen	1 (6,7%)
Bone marrow transplant in 1 CR	7 (47%)
Follow-up	
Relapse	11 (73,3%)
Progression on primary therapy	1 (6,7%)
Median TTR in months	9.9

n - number; CR – complete remission; TTR – time to relapse

At the time of the current intervention, 11 patients had experienced relapse, one had disease progression while on first line therapy, and three patients with metastatic disease were in remission after first line therapy with radiation therapy and chemotherapy. The reason for trofosfamide therapy was relapse in eleven patients, progression on primary therapy in one patient, three patients received treatment because of lack possibilities to primary site surgery (they were treated radiation therapy only). Summarizing, maintenance therapy with oral trofosfamide was administered in 10 patients with no evidence of active disease as a part of radical therapy, in four with progressive disease, and in one with active but stable disease as a palliative regimen (tab. II).

**Table II:** Patient characteristics, response to trofosfamide, and outcome.

Patient nb.	Stage at dlagn.	Patient characteristic according to the treatment	Intention of trofosfamide maintenance therapy	Status of disease before trofosfamide	Nb. of courses	Best response	Toxicity (grade 2-4)	Reason for stopping therapy	Treatment after trofosfamlde	Status (last follow up In years)
1	Met.	1^st^ line	radical	CR	8	CR	renal dysfunction	renal toxicity	-	NED (2.0)
2	Met.	1^st^ line	radical	CR	4	CR	renal dysfunction	renal toxicity	-	NED (1.4)
3	Met.	progression on the 1^st^ line	radical	CR	10	CR	renal dysfunction	renal toxicity	-	NED (4.3)
4	Loc.	relapse	radical	CR	12	CR	none	end of treatment	-	NED (6.3)
5	Loc.	relapse	palliative	PD	2	PD	none	death	-	DOD (7.7)
6	Loc.	relapse	Palliative	PD	2	PD	none	death	-	DOD (9.9)
7	Met.	relapse	radical	CR	6	CR	none	still in treatment	-	NED (3.3)
8	Loc.	relapse	palliative	PD	2	PD	none	progression	VP	DOD (5.7)
9	Met.	1^st^ line	radical	CR	1	CR	leucopenia	marrow toxicity	-	NED (3.2)
10	Met.	relapse	radical	CR	13	CR	none	end of treatment	-	NED (7.6)
11	Met.	relapse	palliative	PD	5	PD	none	death	-	DOD (3.5)
12	Met.	relapse	radical	CR	8	CR	none	end of treatment	-	NED (5.2)
13	Met.	relapse	palliative	SD	8	PD	none	progression	VN/CTX, T	AWD (3.9)
14	Met.	relapse	radical	CR	3	PD	none	progression	TC. HD-IFO	AWD (1.5)
15	Met.	relapse	radical	CR	11	CR	none	end of treatment	-	NED (3.9)

Nb – number; Met – metastases; Loc – localize; VIT–vincristine, irinotecan, temozolomide; PACE – tenipozide, adriamycin, cyclophosphamide, cisplatin; CR – complete response; SD – stable disease; PD–progression disease; DOD-death of disease; NED–no evidence of disease; AWD-alive with disease; T – topotecan; TC–topotecan, cyclophosphamide; VN/CTX–vinorelbine, cyclophosphamide; HD-IFO–high doses ifosfamide; VP–etoposide

A total of 90 cycles were administered, with a median of five cycles per patient. In three patients the treatment was discontinued due to renal toxicity (2 patients developed proteinuria grade 3,1 had glomerular filtration rate (GFR) decrease grade 2). Only one patient developed grade 4 neutropenia and treatment was also discontinued. There were no other significant toxicities (tab. II).

Nine of ten patients in complete remission (CR) at time of trofosfamide are alive with no evidence of disease with a median follow-up of 3.9 years (range 1.4 to 7.6), and one is alive with evidence of disease (TTP was 5.3 months). All patients in progressive disease (PD) at the time of trofosfamide died, median TTP was 1.9 months (range 1.8 to 4.6 months). One patient with stable disease (SD) at the time of trofosfamide is alive with evidence of disease (TTP was 2.8 months). Median time from start of trofosfamide to progression disease in the whole group was 1.9 months (range 1.8 to 4.6 months). The estimated 2-year survival for the whole group was 61%. Treatment, response, and outcome are depicted in table II.

## Discussion

Here we have presented our results with the use of trofosfamide regimen in the management of patients with primary bone Ewing sarcoma and have confirmed the feasibility of this approach. When given after inducing a complete response, we observed a potential benefit in maintaining remission.

The outcome for patients with metastatic Ewing sarcoma remains poor and new treatments are urgently needed [2, 3, 5, 7, 19]. The idea of the use of oral trofosfamide was drawn from experience with soft tissue sarcomas, where promising results have been achieved in patients with extra skeletal Ewing sarcoma. Klingebiel et al. compared the oral maintenance therapy vs. high dose therapy in patients with soft tissue sarcoma (RMS-like tumours). The study included 14 patients below 22 years; patients were treated with two alternating schedules: trofosfamide plus idarubicin versus trofosfamide plus etoposide, for a total of 8 cycles. Six patients underwent bone marrow transplant (BMT). The authors showed that oral maintenance therapy could be a good option for patients with advanced RMS-like tumours. In a second study, Hartman et al. treated 18 adult patients (median age 57 years, range 27-78 years) with advanced pre-treated soft tissue sarcoma (STS), with no objective responses; however, almost half of the patients achieved disease stabilization for half a year. Also other studies have shown promising results and low toxicity in patients with different tumours (adrenocortical tumours [11], advanced metastatic prostate cancer [13], malignant melanoma [12], lymphoma [14] and ovarian carcinoma [20]). Two other studies have described the efficacy of this drug in maintenance therapy for advanced bone and soft tissue sarcomas. Laws et al. reported on two patients with refractory Ewing sarcoma, both received maintenance treatment (trofosfamide together with etoposide) and one of the two patients benefited from the therapy. Another study on adult patients has shown that oral maintenance therapy with trofosfamide for locally advanced or metastatic high-grade soft tissue and bone sarcomas with doses similar to ours (100-150 mg per day) was well-tolerated and seemed to prolong progression-free and overall survival [22]. In our study, patients in CR at the time of trofosfamide, responded well to the regimen (nine of ten patients are alive with no evidence of disease). Furthermore, this group also included heavily pre-treated relapsed patients.

Blomqvist has shown 23 patients with metastatic sarcomas treated with trofosfamide and no severe toxicities were observed. In our study we also have not observed severe toxicity, however, in three patients the treatment was discontinued due to renal toxicity. Thus, it seems that during the trofosfamide treatment patients should be closely monitored for renal complications.

## Conclussions

Treatment with trofosfamide is well-tolerated and could have a role to maintain response in patients with primary bone Ewing sarcoma. Further studies are needed to better define the use of this regimen in the upfront management of those patients.
